# For and against Organ Donation and Transplantation: Intricate Facilitators and Barriers in Organ Donation Perceived by German Nurses and Doctors

**DOI:** 10.1155/2016/3454601

**Published:** 2016-08-15

**Authors:** Niels Christian Hvidt, Beate Mayr, Piret Paal, Eckhard Frick, Anna Forsberg, Arndt Büssing

**Affiliations:** ^1^Research Unit of General Practice, Institute of Public Health, Faculty of Health Sciences, University of Southern Denmark, J. B. Winsløwsvej 9A, 5000 Odense C, Denmark; ^2^Forschungsstelle Spiritual Care, Klinik und Poliklinik für Psychosomatische Medizin und Psychotherapie, Munich School of Philosophy, Kaulbachstraße 31, 80539 Munich, Germany; ^3^Research Centre Spiritual Care, Department of Psychosomatic Medicine and Psychotherapy, The University Hospital Klinikum rechts der Isar, Langerstraße 3, 81675 Munich, Germany; ^4^Hospice Care DaSein, Karlstraße 55, 80333 Munich, Germany; ^5^Department of Transplantation and Cardiology, Skåne University Hospital, 221 85 Lund, Sweden; ^6^Department of Health Sciences, Lund University, P.O. Box 157, 221 00 Lund, Sweden; ^7^Institute of Integrative Medicine, Faculty of Medicine, Witten/Herdecke University, Herdecke, Gerhard-Kienle-Weg 4, 58313 Herdecke, Germany

## Abstract

*Background.* Significant facilitators and barriers to organ donation and transplantation remain in the general public and even in health professionals. Negative attitudes of HPs have been identified as the most significant barrier to actual ODT. The purpose of this paper was hence to investigate to what extent HPs (physicians and nurses) experience such facilitators and barriers in ODT and to what extent they are intercorrelated. We thus combined single causes to circumscribed factors of respective barriers and facilitators and analyzed them for differences regarding profession, gender, spiritual/religious self-categorization, and self-estimated knowledge of ODT and their mutual interaction.* Methods.* By the use of questionnaires we investigated intricate facilitators and barriers to organ donation experienced by HPs (*n* = 175; 73% nurses, 27% physicians) in around ten wards at the University Hospital of Munich.* Results.* Our study confirms a general high agreement with the importance of ODT. Nevertheless, we identified both facilitators and barriers in the following fields: (1) knowledge of ODT and willingness to donate own organs, (2) ethical delicacies in ODT, (3) stressors to handle ODT in the hospital, and (4) individual beliefs and self-estimated religion/spirituality.* Conclusion.* Attention to the intricacy of stressors and barriers in HPs continues to be a high priority focus for the availability of donor organs.

## 1. Introduction

Alone in the USA over an estimated 120.000 people are waiting for a donor organ with 21 patients dying per day due to the deficit [1]. In Germany around 1.000 persons die per year while awaiting organ transplantation [2], with similar numbers for other developed nations, although most of them implement significant national campaigns and other incentives toward an increase in available organs [3–7]. Despite growing numbers of organ donations and transplantations (ODT), ever more people need donor organs due to the increase in metabolic diseases, for example, diabetes and obesity. The progress of modern medicine enables a growing variety of possible transplantations and hence there is an ever increasing gap between the availability of and the need for donor organs [8].

Despite the flagrant need for donor organs, resistance remains among the general public and even among health professionals to ODT. This should not come as a surprise. ODT constitutes a complex ethical and value laden field of interdisciplinary interventions. It is a surgical and medical field that requires the highest scientific standards, but likewise one, where ethics, values, and personal beliefs play an immense role. Not surprisingly, then, extensive research has been done on attitudes to ODT in the general public, in medical students, and in health professionals (HPs) [9–11] often with the explicit aim of investigating whether they “have the knowledge needed to maximize organ donation rates” [12] or “to inform strategies to improve organ donation rates” [13].

Studying the attitudes of HPs has been found to be of particular relevance. Publications addressing psychosocial and ethical issues have shown that despite the obvious need for organ donation, the most important factor hindering ODT is, despite the will of the deceased, the attitude of intensive care unit members to organ donation [9, 14].

On the basis of existing research literature and an expert focus group study (see the following), it became clear that the barriers and facilitators in ODT medicine are multiple and intricate as they relate to ODT knowledge, ethics, stressors, individual beliefs, and religiosity. Hence, the purpose of this study was to investigate to what extent HPs (physicians and nurses) experience such facilitators and barriers in ODT and to what extent they are intercorrelated. We thus intended to combine single causes to circumscribed factors of respective barriers and facilitators to analyze them for differences regarding profession, gender, spiritual/religious self-categorization, and self-estimated knowledge of ODT and to gauge their interaction.

## 2. Participants and Methods

### 2.1. Development of Survey Items

We developed a survey in close collaboration with leading staff of the Bavarian branch of the German Organ Transplantation Foundation (DSO) to map personal values and beliefs with attitudes toward ODT. After an extensive literature review, we conducted a multiprofessional focus group discussion with 15 experts in the field of ODT (Mayr et al., in preparation) to identify various facilitators and barriers in ODT. The focus group discussion was recorded and transcribed verbatim and analyzed using Thematic Content Analysis [15], to identify main categories and subthemes, which should be addressed in the intended survey.

This review and qualitative development process led to the identification of five relevant themes for the experience of barriers and facilitators in ODT: (1) knowledge of ODT, (2) ethical appraisal of ODT, (3) ethical arguments favoring ODT in dialogue with relatives, (4) stressors, and (5) belief barriers.

### 2.2. Validation of the Survey Items

The above-stated categories were the primary source for the items used in the current survey. The postulated questions were carefully discussed in the preliminary expert rounds and then tested among medical students and professionals. The optimized version was tested among further healthcare professionals by using the think-aloud protocols [16]. The interviews lasted from 30 minutes to an hour and helped us to refine some further misunderstandings and remove unclear statements from the final draft. The saturation was achieved after conducting 9 interviews. The final version of the survey was conducted in summer 2014.

Items were scored on a 4-point scale ranging from strong agreement (1) to strong disagreement (4) or similar phrasing, ranging from “applies exactly” (1) to “does not apply at all” (4). Thus, the higher the scores, the stronger the disagreement.

During the next step of the validation process, we exploratively tested the factorial structure of the rather heterogeneous item topics and finally the internal reliability (Cronbach's coefficient *α*) of putatively sound factors in a larger sample of 175 HPs. When such factors were identified (principal component analysis using varimax rotation with Kaiser's normalization), we tested different theoretically plausible structures and subsequently eliminated those items which loaded weekly on the respective factor (<.05), those items which would load strongly on two concurrent factors, or those with a weak item to scale correlation. It was not the intention to design an instrument but to test differences in the attitudes of the health care professionals. In fact, none of the scales were designed as a specific construct (apart from the general topic), and thus some of the identified factors are less balanced with respect to item number. Apart from face validity, for this study we had no external measures to analyze construct validity.

### 2.3. Statistics

Data were entered by scanning the completed paper surveys in the scanning software* ZENSUS* developed by Blubbsoft. Descriptive statistics, internal consistency (Cronbach's *α*), and factor analyses as well as analyses of variance and first-order correlations were computed with SPSS 22.0. Due to the exploratory character of the study, the level of significance was set at  .05.

The study obtained ethics approval (#383-12/2014) was gotten from the Ethics Committee of Ludwig Maximilian University of Munich.

## 3. Results

A total of 293 paper questionnaires were distributed in around ten wards at University Hospital in Munich to both physicians and nurses working in various ways and to different degrees with ODT in medical and surgical departments. The survey was introduced to the team members of every participating ward by members of the research team. The response rate was 64% (*n* = 175). Responding HPs were nurses (73%) and physicians (27%); 71% were female. Eleven questionnaires were discarded, because respondents had chosen not to fill in demographic data. Sociodemographic and employment data are presented in Table 1.

In total, 45% were Catholics, 21% Protestants, 4% had other affiliations, and 30% were not affiliated. With respect to their religious and/or spiritual self-categorization, 28% regard themselves as both religious* and* spiritual (R+S+), 12% religious but* not* spiritual (R+S−), 7% spiritual but* not* religious (R−S+), and 53% neither religious* nor* spiritual (R−S−). This quadropartition has been found to be a viable way of identifying different types of R/S [17]. With respect to this self-categorization, there were no significant differences between women and men (data not shown).

Within the sample, 41% believed in life after death and 34% did not, whereas 25% were undecided. In trend, more women (47%) than men (27%) were convinced (*p* = .07), while there were no significant differences with respect to profession (*p* = .78). Interestingly, a significantly larger percentage of Catholics believed in life after death (54%) than Protestants in the sample among whom only 38% believed in life after death (figures significant).

### 3.1. Knowledge Barriers and Willingness to Donate Own Organs

Within the sample, 92% stated to be adequately informed about the legal regulatory aspects of ODT and 96% about brain death signs. The few who did not consider themselves satisfactorily informed about the regulatory aspects were mainly found in the group of nurses (9.5% of nurses and 2.2% of physicians; *p* = .092). Further, 67% of HPs agreed with the regulatory aspects of ODT, that is, 54% of physicians and 71% of nurses (*p* = .030), others obviously not.

When asked about their own consent to become an organ donor after death, a vast majority of the HPs agreed to donate their organs (77%) and tissue (such as the cornea or heart valves (71%)). There were no significant differences for gender, profession, and spiritual/religious self-categorization (data not shown).

We next intended to combine specific topics (either facilitators or barriers) addressed with different single items to specific factors and tested first their internal reliability before we would address differences between HPs with respect to these topics.

### 3.2. Reliability of Factors Related to Specific ODT Topics

#### 3.2.1. Ethical Appraisals of ODT

To address ethical barriers HPs perceived in ODT, the respective items were condensed to specific factors. However, the internal reliability of these six items was rather weak (Table 2). Exploratory factor analysis pointed to two subconstructs, one with four items (alpha = .67) and one with two items (alpha = .47). Only the first scale (*ethical barriers to ODT*) might be used for further analyses, while the quality of the second is too weak. Within the first *i* factor, the lowest scores were found for “justice in the distribution of organs” (indicating agreement) and the highest for “handling of the personal convictions of colleagues” (indicating disagreement).

#### 3.2.2. Ethical Facilitators to ODT in the Dialogue with Relatives

In the developmental phase we found that beliefs and values favoring ODT were most clearly formulated as arguments for ODT when HPs conversed respectfully with people who reflected whether they should release the body of their brain dead relative for ODT. Hence, HPs were asked whether they believed it was acceptable to propose arguments to relatives in favor of ODT and what would be viable arguments proposed in such conversations.

The facilitating arguments were tested for their reliability. As shown in Table 3, the respective seven items had a satisfactory internal reliability (alpha = .77), with two subconstructs which would explain 61% of variance. The first construct,* personal ethical facilitators* (alpha = .73), regarded ethical arguments that relatives could see for themselves, whereas the second construct,* concrete altruistic effects* (alpha = .72), regarded advantages others could have of the act of giving the relatives' organs. The three items addressing* concrete altruistic effects* scored lower than the* personal ethical facilitators* (with the highest disagreement score for the item stating that “your consent is an ethical duty”).

#### 3.2.3. Stress Barriers in ODT

Next we asked for the stress barriers of HPs which were addressed with six items (Table 4). Exploratory factor analysis pointed to two subconstructs, one with four items and satisfactory internal reliability (*medical reasons*; alpha = .74) and one with two items and poor internal reliability (*team reasons*; alpha = .33). Only the first scale might be used for further analyses. Here, the strongest disagreement was found for “care for relatives” as putative stressful barrier, while “acceptance of brain death as death of a human being” was considered less of a stressful barrier.

#### 3.2.4. Belief Barriers in ODT

We asked respondents which representations in dying and death could constitute a barrier for ODT from their personal perspective and from the assumed perspective of relatives. Four questions related to the immanent, earthly life, whereas four items related to the transcendent and to the afterlife. We differentiated perceived own ODT barriers (Table 5(a)) and those assumed for the relatives (Table 5(b)).

As shown in Table 5(a), the eight items addressing personal perception of ODT barriers had a good internal reliability (alpha = .88) and two subconstructs. Because the item addressing the “wish to be buried as a whole” would load on both factors, it was eliminated from the item pool. Factor one would thus address* transcendent barriers: protection of the soul* (alpha = .87) and factor two* immanent barriers: affection of the physical body* (alpha = .79). The mean scores of both subscales are similar. The highest scores (indicating disagreement) were found for the “wish that the body should resurrect integrally.” The same structure was found when HPs considered ODT barriers of relatives (Table 5(b)). Here, the item addressing the “wish to be buried whole” would load best on the factor* immanent barriers*, but considerably also on the factor* transcendent barriers*, and was thus eliminated from that item pool, too.

### 3.3. Correlations between Facilitators and Barriers

With these factors we analyzed whether or not the addressed facilitators and barriers were associated in any way. As shown in Table 6, both factors addressing ODT arguments to be communicated to relatives were either not at all or only marginally associated with ODT barriers. Thus, these aspects have to be seen as independent dimensions. Neither the stress barriers nor the ethical issues showed any significant association with the other factors.

### 3.4. Facilitators and Barriers within the Sample

Generally, we can show that, with respect to ODT arguments communicated to relatives,* concrete altruistic effects* receive higher agreement (lower scores) than* personal ethical facilitators* (higher scores) (Table 7). Although there were no significant differences with respect to gender, nurses scored higher disagreement for* personal ethical facilitators* (*F* = 4.6, *p* = .034; Cohen's *d* = 0.40) and* concrete altruistic effects* (*F* = 5.9, *p* = .016; Cohen's *d* = 0.44). The effects are rather small. However, the SpR self-categorization has a significant effect only on the* personal ethical facilitators*, which scored higher on disagreement in R−S− HPs (*F* = 8.5, *p* = .004; Cohen's *d* = 0.49). The high scores on the own perception of transcendent and also immanent barriers scales indicate HPs' general disagreement, without any significant difference for gender, profession, or SpR self-categorization. Particularly the assumed immanent ODT barriers of relatives scored lower than transcendent barriers (with similar disagreement level for one's own perception of ODT barriers), suggesting that HPs would particularly disagree with the protection of the soul (*transcendent barrier*) as an ODT barrier when compared to the affection of the physical body (*immanent barrier*).

Medical reasons as stress barriers are of lower relevance in the sample, particularly for women (*F* = 11.4, *p* < .0001; Cohen's *d* = 0.59) and nurses (*F* = 16.2, *p* < .0001; Cohen's *d* = 0.72) which had the highest scores indicating disagreement. The effect sizes are moderate. HP's SpR attitude had no significant influence.

There were no significant differences in the perception of ethical barriers to ODT for gender, profession, or SpR self-categorization.

### 3.5. Facilitators and Barriers in HPs Who Would Agree to Own ODT

When the HPs were categorized for their willingness to serve as an organ donor, we saw significant differences: those who do not wish to donate their own organs showed stronger disagreement to communicate* concrete altruistic effects* (*F* = 7.4, *p* = .007; Cohen's *d* = 0.55) or* personal ethical facilitators* (*F* = 4.6, *p* = .034; Cohen's *d* = 0.44) as ODT arguments when compared to those would agree. The effect size is moderate and small, respectively. With respect to the belief barriers, there were no significant differences.

Few who do not feel adequately informed about the regulatory aspects of ODT had stronger disagreement for* personal ethical facilitators* (*F* = 7.3; *p* = .008; Cohen's *d* = 0.78) and* concrete altruistic effects* (*F* = 6.6, *p* = .011; Cohen's *d* = 0.76) to be communicated to relatives. The effect sizes are moderate. With respect to the belief barriers, there were no significant differences, too.

## 4. Discussion

### 4.1. For and against ODT

The vast majority of HPs were in favor of ODT. In fact, most consented to donate their own organs (77%), they agreed that the lack of organs is an ethical problem (88%), and they found it acceptable to propose arguments to relatives in favor of ODT (78%). These findings confirm existing research indicating that HPs working in ODT are generally highly motivated [11, 18].

However, the HPs in our study also identified various types of interwoven barriers and facilitators in ODT, confirming the fact that ODT is a difficult medical and ethical field in which to navigate. Interestingly, we saw a tendency that HPs tended to disagree with the barriers that they themselves considered significant factors barring ODT. When communicating with relatives, the majority of HPs found it rather acceptable to propose concrete altruistic arguments that have formerly been found associated with high ODT advocacy [11, 19].

#### 4.1.1. Knowledge Barriers and Facilitators

The HPs in our study reported a high degree of being well informed of legislative aspects of ODT (92%) and the signs of brain death (96%). This does, however, not entail that all HPs agree with the legislative aspects of ODT. In fact, only 67% of HPs in our sample agreed (54% physicians and 71% nurses), suggesting a potential conflict encircling the existing legal practices. The few who did not feel adequately informed about regulatory aspects of ODT tended in general to be less willing to propose arguments in favor of ODT in the dialogue with relatives, suggesting low commitment to ODT advocacy. 

Our findings thus confirm former research indicating lack of knowledge regarding ODT as one of the primary potential barriers to ODT in the public [20] and in HPs as well [21–25]. It has been found to be of particular importance as HPs approach donor families [26]. The range of barriers in physicians and nurses related to specific knowledge of ODT has been described as perception of organ and tissue transplantation as an experimental procedure, knowledge about criteria for potential donors, request policies and procedures, and understanding and explaining brain death to relatives [25].

As mentioned, most HPs in our sample agreed to donate their own organs, something formerly found to be generally correlated with a high commitment to ODT advocacy [19, 25]. This is confirmed as well in our sample as those who would donate organs tend to be more in favor of providing both above-mentioned types of arguments for ODT in dialogue with relatives than those who do not wish to donate own organs.

#### 4.1.2. Ethical Barriers and Facilitators

In our study, HPs saw significant ethical barriers to ODT particularly in the “justice” of the “distribution of organs.” This confirms existing research in the field suggesting that HPs consider legal, ethical, and value laden questions in ODT to constitute significant barriers in HPs to ODT [27, 28].

With regard to the ethical facilitators in ODT, the majority of HPs in our study agreed that it was acceptable to propose ethical arguments in the dialogue with relatives of potential donors that as mentioned has been found associated with high ODT advocacy. We found two ethical constructs that HPs considered important in the dialogue with relatives:* personal ethical facilitators* regarded arguments that relatives could relate as relevant to themselves, whereas* concrete altruistic effects* entailed advantages other people could gain from the relatives' decision to agree with the donation of their loved one's organs. The HPs generally favored* concrete altruistic effects* over* personal ethical facilitators*. Ethical arguments for ODT, such as ODT being an act of charity or responsibility of fellow human beings as found in our study, have been proposed in favor of monetary or legal incentives [29–32]. Thus, a study by Jasper et al. found that the vast majority of HPs preferred the policy of altruistic organ donation from a moral perspective over different types of possible incentives to donor families, although they also agreed that such altruistic policy was not sufficiently effective [33].

#### 4.1.3. Stress Barriers

The HPs in our sample identified differentiated stress barriers to ODT in their daily clinical work. HPs considered “care for the relatives” a lesser stressful barrier than “acceptance of brain death as death of a human being.” The strongest variance was found for nurses (and thus women, too) who disagreed that* suggested medical reasons* constituted stress barriers. One might assume that they are more involved in the care of donors and their relatives than with making clinical ODT decisions, and thus they do not see medical reasons as strong arguments against ODT.

This should come as no surprise on the basis of international research. Important barriers and facilitators are found in the perceived stress and coping resources in handling ODT. Such stress and resources have not only been identified by relatives of potential donors and by those waiting for an organ [34, 35] but are experienced as well by HPs in ODT. For instance, Hibbert identified multiple stressors experienced by nurses, such as the threat of the dying patient and the inconsistent commitment of physicians to organ donation [36], but she also identified their work as meaningful to them and found coping resources for handling such stress in gaining control over emotions, distancing oneself, and taking timeout [37].

#### 4.1.4. Belief Barriers and Facilitators

In our study, we identified two constructs of barriers: first, “transcendent barriers: protection of the soul,” that is, barriers relating to transcendent, spiritual/religious notions working against ODT advocacy; second, “immanent barriers: affection of the physical body.” In general, the HPs tended to see belief barriers to a lesser degree for themselves than for relatives, particularly immanent beliefs (such as “that ODT violates the body”) as an assumed barrier for relatives. HPs tended to rather disagree that transcendent beliefs (such as “the wish that the body should resurrect integrally”) constituted barriers to ODT.

As mentioned, HPs tended to feel discomfort proposing* personal ethical facilitator* arguments; interestingly R−S− HPs were more reluctant to propose such arguments to relatives than their R+S+ counterparts, which might suggest a correlation between R+S+ and ODT advocacy. There was no significant difference between R−S− and R+S+ with respect to* concrete altruistic effects* as arguments just as personal R+S+ had no significant effect on their own belief, stress, or ethical barriers to ODT.

A large bulk of research has centered on how religion/spirituality can entail both barriers and facilitators to ODT [13, 38–40]. Today, there seems to be a growing tendency for religious/spiritual arguments to favor ODT rather than the opposite. Thus, what surprised Jasper et al. in their study was that religion was offered far more often as a rationale for wanting to help sick people through organ donation than it was for not wanting to donate organs [33]. Likewise, more and more religious leaders recommend giving one's organs as an act of charity [41, 42]. Along the same vein, Abidin et al. even propose “increasing (ODT) awareness of the public through religion” [14].

Moreover, it has been shown that religious beliefs impact the concrete practice of various fields of medicine such as general practice [43], psychiatry [44, 45], gynaecology [46], and end-of-life-care [47]. This research documents how such beliefs may have significant impact on the practice of medicine, including ODT. In our study, we find hints that spiritual/religious attitudes were partly and specifically associated with ODT advocacy.

### 4.2. Multiplicity and Intricacy of Facilitators and Barriers

International research indicates strong correlation between the various facilitators and barriers in ODT. Thus, Irving and coworkers point to the intricacy of multiple barriers and facilitators and write that “intractable factors, such as religion and culture, are often tied in with more complex issues such as a distrust of the medical system, misunderstandings about religious stances and ignorance about the donation process” [13]. Our study lends limited evidence to this insight, as the interdependency of ODT factors (Table 6) and different ODT variables (Table 7) only show some interactions.

Such multiplicity and intricacy have been found important in other studies and are evident in the professional setting, even from a structural, systemic perspective, as ODT entails a complex multiprofessional, ethical interaction: (1) it generally depends on the cooperation of various hospitals, departments, professions, and organ allocation institutions; (2) organ donation depends on HP interaction with patients, potential organ donors, and their relatives. This multidimensional interaction holds many inherent barriers against successful ODT. In such a complex field, there are no singular causes. In line with* system*,* process*, and* force field theory*, causes are interdependent and act upon each other [48]. The same goes for psychological behaviour [49] and social interaction [48, 50]. Thus, in a “force field” as complex as ODT, it is important to study and clinically to consider not merely the impact of single but rather numerous intricate causes for the lack of organs today in order to select adequate strategies to enhance the availability of organs for ODT.

## 5. Limitations

The study population is small and HPs were recruited in a few departments of the University Hospital in Munich only and with a response rate of 64%. Although we do not assume the data as representative of ODT HPs in general, we at least can add further important aspects to the general discussion. For future studies larger sample sizes and inclusion of other regions of Germany (with their specific cultural settings) should be included. Due to the cross-sectional design of this exploratory study, causal interpretations are not possible.

## 6. Conclusion

This study confirms a general high agreement with the importance of ODT among ODT HPs. Nevertheless, we identified both facilitators and barriers in the following fields that impact each other: (1) knowledge of ODT and willingness to donate own organs, (2) ethical delicacies in ODT, (3) stressors to handle ODT in the hospital, and (4) individual beliefs and self-estimated religion/spirituality. Thus we found that ODT constitutes a medically and ethically complex and intricate field of medical intervention and that continuous optimization of HPs' knowledge of ODT is of relevance for their own perception of barriers and facilitators through education and continued learning. Continued learning concerning specific knowledge of brain death has decreased the experienced ethical and practical barriers that the notion of brain death constitutes ODT [51]. Trials on the efficiency of increasing knowledge on the facts and needs of ODT in the general public and in health professionals through public campaigns and HPs continued learning raises positive attitudes to ODT, including the will to become a donor [52].

Recognition and articulation of personal beliefs and convictions in both relatives and HPs are likewise of high relevance for ODT, although often considered a personal matter and not one of medical discourse. Insights and experiences could be brought to ODT from the palliative field, where the actual handling of such intricate ethical and spiritual values and beliefs is very much part of medical attention, even in a rather secularized European setting. Our study suggests that actively addressing the perceived belief barriers in ODT through interdisciplinary teamwork including both HPs but also psychologists and chaplains may continue to enhance a favourable ODT culture.

Finally, recognizing the intricacy of barriers and facilitators in ODT may contribute to the facilitation of ODT in avoiding blind spots in the continued efforts to help more people survive due to better availability of organs for transplantation.

## Figures and Tables

**Table 1:** Characterization of enrolled persons (*n* = 175).

*Age (years)*	33.9 ± 11.1

*Gender (%)*	
Women	71
Men	29

*Family status (%)*	
With partner	55
Single	42
Divorced/widowed	3

*Confession (%)*	
Catholic	45
Protestant	21
Other	4
None	30

*SpR self-categorization (%)*	
R+S+	28
R+S−	12
R−S+	7
R−S−	53

*Profession (%)*	
Physicians	27
Nurses	73

*Employment (%)*	
Full time	86
Part time	14

*Hospital (%)*	
With TX unit	92.5
Without Tx unit/other	7.5

*Working area (%)*	
Donors	26
Donees	46
Both	20
Neither nor	8

*Perceived health impairment (1–11) *	
Physical	4.0 ± 2.8
Mental	3.8 ± 2.7

**Table 2:** Mean values, reliability, and factor analysis of item addressing the perception of ethical issues.

Factors and items	Mean value (score 1–4)^*∗*^	SD	Corrected item-total correlation	Alpha if item is deleted (*α* = .683)	Loading factor 1	Loading factor 2
*Factor 1: ethical barriers to ODT* (eigenvalue 2.2; 38% explained variance; alpha = .67)						
Handling of the personal convictions of colleagues	2.40	0.83	.395	.648	.781	
Respect for the individual problems of patients/relatives	2.16	0.76	.426	.638	.726	
Transparency of the system	2.01	0.89	.548	.591	.634	.406
Justice in the distribution of organs	1.87	0.83	.445	.631	.533	.403

*Factor 2: external ethical issues in ODT* (eigenvalue 1.0; 17% explained variance; alpha = .47^*∗∗*^)						
Scandals in transplantation medicine	1.71	0.77	.335	.667		.773
Lack of organs	1.70	0.76	.326	.669		.737

Extraction of the main components (eigenvalue > 1); varimax rotation with Kaiser's normalization.

Rotation is converged in 3 iterations. Both factors explain 55% of variance.

^*∗*^Scores range from 1 (agreement) to 4 (disagreement).

^*∗∗*^Scale is not suited to be used.

**Table 3:** Mean values, reliability, and factor analysis of item addressing the agreement to consider facilitating ODT arguments with relatives.

Factors and items	Mean value (score 1–4)^*∗*^	SD	Corrected item-total correlation	Alpha if item is deleted (*α* = .774)	Loading factor 1	Loading factor 2
*Factor 1: personal ethical facilitators* (eigenvalue 2.9; 42% explained variance; alpha = .73)						
Your consent could be a source of meaning in your own life	2.78	0.94	.603	.723	**.841**	
Your consent would be an act of charity	2.75	0.99	.569	.730	**.721**	
Your consent is an ethical duty	3.50	0.77	.355	.771	**.710**	
The death of the diseased would have a purpose	2.70	1.03	.491	.749	**.555**	.323

*Factor 2: concrete altruistic effects* (eigenvalue 1.3; 18% explained variance; alpha = .72)						
Your consent can save the life of another person	1.47	0.74	.462	.754		**.880**
Your consent can do good	1.69	0.85	.509	.744		**.833**
You might come to a point where you yourself could be in need of a transplantation	1.98	0.99	.498	.746	.333	**.610**

Extraction of the main components (eigenvalue > 1); varimax rotation with Kaiser's normalization.

Rotation is converged in 3 iterations. Both factors explain 61% of variance.

^*∗*^Scores range from 1 (agreement) to 4 (disagreement).

**Table 4:** Mean values, reliability, and factor analysis of item addressing stress barriers in the care of potential donors with brain death.

Factors and items	Mean value (score 1–4)^*∗*^	SD	Corrected item-total correlation	Alpha if item is deleted (*α* = .696)	Loading factor 1	Loading factor 2
*Factor 1: stress barriers: medical reasons* (eigenvalue 2.4; 41% explained variance; alpha = .74)						
Spinal or vegetative reflexes, such as lazarus signs	2.56	0.87	.473	.640	.801	
Continuation of intensive care, despite established brain death	2.29	0.92	.559	.607	.709	.326
Acceptance of brain death as death of a human being	2.02	0.83	.607	.595	.686	.427
Care for relatives	3.03	0.81	.376	.671	.654	

*Factor 2: stress barriers: team reasons* (eigenvalue 1.0; 17% explained variance; alpha = .33^*∗∗*^)						
Overwork/having to take the position of a colleague who does not take part in ODT	2.12	0.82	2.74	.703		.867
Overwork/having to take the position of a colleague who does not take part in ODT	2.42	0.86	.285	.699		.565

Extraction of the main components (eigenvalue > 1); varimax rotation with Kaiser's normalization.

Rotation is converged in 3 iterations. Both factors explain 57% of variance.

^*∗*^Scores range from 1 (agreement) to 4 (disagreement).

^*∗∗*^Scale is not suited to be used.

**(a):** 

Factors and items	Mean value (score 1–4)^*∗*^	SD	Corrected item-total correlation	Alpha if item is deleted (*α* = .878)	Loading factor 1	Loading factor 2
*Factor 1: transcendent barriers: protection of the soul* (eigenvalue 4.3; 53% explained variance; alpha = .87)						
The wish that the body should resurrect integrally	3.07	0.99	.686	.858	**.885**	
Belief in reincarnation, rebirth, karma, or similar	2.96	0.99	.635	.863	**.821**	
The wish to arrive intact in the afterlife	2.67	1.06	.705	.855	**.783**	.310
That the soul prevails in the body beyond established death	2.92	1.00	.694	.857	**.749**	
The wish to be buried whole^*∗∗*^	2.21	1.02	.666	.859	.527	.512

*Factor 2: immanent barriers: affection of the physical body* (eigenvalue 1.2; 15% explained variance; alpha = .79)						
That ODT violates the body	2.75	1.02	.556	.871		**.873**
That the corpse would be blemished	2.30	0.99	.663	.860		**.832**
That the process of death is not complete with brain death	2.21	1.13	.521	.867		**.680**

Extraction of the main components (eigenvalue > 1); varimax rotation with Kaiser's normalization.

Rotation is converged in 3 iterations. Both factors explain 68% of variance.

^*∗*^Scores range from 1 (agreement) to 4 (disagreement).

^*∗∗*^Without item “wish to be buried whole.”

**(b):** 

Factors and items	Mean value (score 1–4)^*∗*^	SD	Corrected item-total correlation	Alpha if item is deleted (*α* = .874)	Loading factor 1	Loading factor 2
*Factor 1: transcendent barriers: protection of the soul* (eigenvalue 4.3; 54% explained variance; alpha = .93)						
The wish that the body should resurrect integrally	2.34	0.89	.796	.840	**.896**	
Belief in reincarnation, rebirth, karma, or similar	2.38	0.90	.731	.848	**.878**	
The wish to arrive intact in the afterlife	2.22	0.83	.776	.843	**.875**	
That the soul prevails in the body beyond established death	2.35	0.90	.758	.844	**.853**	

*Factor 2: immanent barriers: affection of the physical body* (eigenvalue 1.4; 18% explained variance; alpha = .75)						
That the corpse would be blemished	1.71	0.67	.585	.865		**.845**
That the process of death is not complete with brain death	1.49	0.66	.321	.886		**.770**
That ODT violates the body	2.11	0.91	.535	.871		**.752**
The wish to be buried whole^*∗∗*^	1.87	0.75	.563	.866	.466	.516

Extraction of the main components (eigenvalue > 1); varimax rotation with Kaiser's normalization.

Rotation is converged in 3 iterations. Both factors explain 71% of variance.

^*∗*^Scores range from 1 (agreement) to 4 (disagreement).

^*∗∗*^Without item “wish to be buried whole.”

**Table 6:** Correlations between the tested factors.

	ODT arguments to be communicated to relatives		Own perception of ODT barriers		Putative ODT barriers of relatives		Stress barriers: medical reasons	Ethical barriers to ODT
	Personal ethical facilitators	Concrete altruistic effects	Transcendent barriers	Immanent barriers	Transcendent barriers	Immanent barriers		
*ODT arguments to be communicated to relatives *								
Personal ethical facilitators	1,000	,463^*∗∗*^	−,029	,142	,054	,223	−,003	.012
Concrete altruistic effects		1,000	−,131	,059	,003	,219	,015	−,013

*Putative own ODT barriers*								
Transcendent barriers: protection of the soul			1,000	,512^*∗∗*^	,376^*∗∗*^	,151	−,091	−,025
Immanent barriers: affection of the physical body				1,000	,193	,487^*∗∗*^	−,148	−,004

*Putative ODT barriers of relatives*								
Transcendent barriers: protection of the soul					1,000	,381^*∗∗*^	−,023	,117
Immanent barriers: affection of the physical body						1,000	−,110	,166

*Stress barriers: medical reasons*								.010

^*∗∗*^ *p* < .001 (Spearman rho).

**Table 7:** Mean values.

		ODT arguments to be communicated to relatives		Own perception of ODT barriers		Assumed ODT barriers of relatives		Stress barriers: medical reasons	Ethical barriers to ODT
		Personal ethical facilitators	Concrete altruistic effects	Transcendent barriers	Immanent barriers	Transcendent barriers	Immanent barriers		
All	Mean	2.92	1.72	2.89	2.41	2.33	1.76	2.46	2.10
	SD	0.69	0.72	0.87	0.89	0.80	0.62	0.67	0.59

*Adequately informed about regulatory aspects of ODT*									
Yes (92%)	Mean	2.87	1.68	2.91	2.41	2.32	1.76	2.43	2.12
	SD	0.68	0.67	0.86	0.89	0.79	0.59	0.66	0.56
No (8%)	Mean	3.40	2.21	2.63	2.46	2.46	1.74	2.79	1.90
	SD	0.65	0.98	0.97	0.98	0.97	0.94	0.73	0.92

*F* value		**7,3**	6,6	1,2	0,0	0,3	0,0	3,5	1,6
*p* value		**.008** ^1^	.011^2^	n.s.	n.s.	n.s.	n.s.	.064	n.s.

*Agreement to own ODT*									
Yes (78%)	Mean	2.86	1.64	2.91	2.44	2.29	1.73	2.44	2.15
	SD	0.71	0.67	0.89	0.93	0.82	0.60	0.65	0.57
No (22%)	Mean	3.16	2.03	2.86	2.34	2.43	1.80	2.53	1.94
	SD	0.59	0.82	0.80	0.81	0.77	0.68	0.73	0.66

*F* value		4.6	**7.4**	0.1	0.3	0.7	0.4	0.5	3.5
*p* value		.034^3^	**.007** ^4^	n.s.	n.s.	n.s.	n.s.	n.s.	.062

*SpR self-categorization*									
R−S− (53%)	Mean	3.07	1.79	2.91	2.44	2.21	1.75	2.47	2.15
	SD	0.68	0.71	0.96	0.94	0.83	0.67	0.63	0.62
R+S+ (47%)	Mean	2.74	1.63	2.90	2.39	2.48	1.78	2.45	2.05
	SD	0.68	0.68	0.74	0.84	0.73	0.56	0.70	0.55

*F* value		**8.5**	1.9	0.0	0.1	4.4	0.1	0.0	1.3
*p* value		**.004** ^5^	n.s.	n.s.	n.s.	.037^6^	n.s.	n.s.	n.s.

*Gender*									
Women (71%)	Mean	2.89	1.71	2.87	2.41	2.29	1.72	2.58	2.07
	SD	0.69	0.72	0.87	0.89	0.78	0.60	0.63	0.56
Men (29%)	Mean	2.98	1.76	2.95	2.43	2.43	1.84	2.20	2.20
	SD	0.70	0.72	0.88	0.92	0.86	0.66	0.68	0.66

*F* value		0.5	0.2	0.3	0.0	1.0	1.1	**11.4**	1.7
*p* value		n.s.	n.s.	n.s.	n.s.	n.s.	n.s.	**<.0001** ^7^	n.s.

*Profession*									
Physicians (26%)	Mean	2.73	1.50	2.93	2.36	2.33	1.64	2.13	2.22
	SD	0.66	0.62	0.85	0.91	0.85	0.55	0.65	0.65
Nurses (73%)	Mean	3.00	1.81	2.88	2.43	2.33	1.80	2.59	2.06
0.60	SD	0.69	0.73	0.88	0.89	0.78	0.64	0.64	0.57

*F* value		4.6	5.9	1.6	2.0	0.0	2.0	**16.2**	2.3
*p* value		.034^8^	.016^9^	n.s.	n.s.	n.s.	n.s.	**<.0001** ^10^	n.s.

^*∗*^Scores range from 1 (agreement) to 4 (disagreement). Thus, the higher the scores are, the more the HPs would disagree.

^1^ *Cohen's d* = *0.78;*

^2^ *Cohen's d* = *0.76;*

^3^Cohen's *d* = 0.44;

^4^ *Cohen's d* = *0.55;*

^5^Cohen's *d* = 0.49;

^6^Cohen's *d* = 0.34;

^7^ *Cohen's d* = *0.59;*

^8^Cohen's *d* = 0.40;

^9^Cohen's *d* = 0.44;

^10^ *Cohen's d* = *0.72*.

## Abbreviations

ODT: Organ donation and transplantation
HP: Health professionals.
